# Content and delivery preferences for information to support the management of high blood pressure

**DOI:** 10.1038/s41371-022-00723-8

**Published:** 2022-08-10

**Authors:** N. Chapman, F. Z. Marques, D. S. Picone, A. Adji, B. R. S. Broughton, Q. N. Dinh, G. Gabb, G. W. Lambert, A. S. Mihailidou, M. R. Nelson, M. Stowasser, M. Schlaich, M. G. Schultz, J. P. Mynard, R. E. Climie

**Affiliations:** 1 Menzies Institute for Medical Research, University of Tasmania, Hobart, TAS Australia; 2 Hypertension Research Laboratory, School of Biological Sciences, Monash University, Melbourne, VIC Australia; 3 Heart Failure Research Laboratory, Baker Heart and Diabetes Institute, Melbourne, VIC Australia; 4 Victor Chang Cardiac Research Institute/ St Vincent’s Hospital, Sydney, NSW Australia; 5 Biomedicine Discovery Institute, Monash University, Melbourne, VIC Australia; 6 Centre for Cardiovascular Biology and Disease Research, Department of Physiology, Anatomy and Microbiology, La Trobe University, VIC Melbourne, Australia; 7 Cardiology Department, Southern Adelaide Local Health Network, Adelaide, SA Australia; 8 Acute and Urgent Care, Central Adelaide Local Health Network, Adelaide, SA Australia; 9 College of Medicine and Public Health, Flinders University, Adelaide, SA Australia; 10 Department of Medicine, Faculty of Health Science, University of Adelaide, Adelaide, SA Australia; 11 Iverson Health Innovation Research Institute, Swinburne University of Technology, Hawthorn, VIC Australia; 12 Department of Cardiology & Kolling Institute, Royal North Shore Hospital, St Leonards, 2065 NSW Australia; 13 Faculty of Medicine & Health Sciences, Macquarie University, Sydney, NSW Australia; 14 Endocrine Hypertension Research Centre, University of Queensland Diamantina Institute, Princess Alexandra Hospital, Brisbane, QLD Australia; 15 Dobney Hypertension Centre, Medical School—University of Western Australia, Perth, TAS Australia; 16 Royal Perth Hospital Unit, Perth, WA Australia; 17 Heart Research, Murdoch Children’s Research Institute, Melbourne, VIC Australia; 18 Department of Paediatrics, University of Melbourne, Melbourne, VIC Australia; 19 Department of Biomedical Engineering, University of Melbourne, Melbourne, TAS Australia; 20 Sports Cardiology Lab, Clinical Research Domain, Baker Heart and Diabetes Institute, Melbourne, VIC Australia

**Keywords:** Risk factors, Lifestyle modification, Medical research

## Abstract

Blood pressure(BP) management interventions have been shown to be more effective when accompanied by appropriate patient education. As high BP remains poorly controlled, there may be gaps in patient knowledge and education. Therefore, this study aimed to identify specific content and delivery preferences for information to support BP management among Australian adults from the general public. Given that BP management is predominantly undertaken by general practitioners(GPs), information preferences to support BP management were also ascertained from a small sample of Australian GPs. An online survey of adults was conducted to identify areas of concern for BP management to inform content preferences and preferred format for information delivery. A separate online survey was also delivered to GPs to determine preferred information sources to support BP management. Participants were recruited via social media. General public participants (*n* = 465) were mostly female (68%), >60 years (57%) and 49% were taking BP-lowering medications. The management of BP without medications, and role of lifestyle in BP management were of concern among 30% and 26% of adults respectively. Most adults (73%) preferred to access BP management information from their GP. 57% of GPs (total *n* = 23) preferred information for supporting BP management to be delivered via one-page summaries. This study identified that Australian adults would prefer more information about the management of BP without medications and via lifestyle delivered by their GP. This could be achieved by providing GPs with one-page summaries on relevant topics to support patient education and ultimately improve BP management.

## Introduction

High blood pressure (BP) is the major cause of non-communicable disease and death globally; in Australia, 34% of adults have hypertension (BP > 140/90 mmHg) [1]. While approaches to lower high BP such as lifestyle changes and medications are widely available and cost-effective in Australia, only 1 in 3 Australians with high BP have their BP controlled, which suggests management of high BP is inadequate as BP remains >140/90 mmHg [1]. Thus, high BP continues to be an important risk factor contributing to the high burden and healthcare costs of heart disease and stroke in Australia [2].

Interventions to optimise BP control among patients have been shown to be more effective when accompanied by appropriate patient education [3]. Patient education can be delivered via a variety of methods including interactive sessions, printed materials, online education sessions and peer-support. Effective patient education can support BP management by improving medication adherence and promoting lifestyle modification [4]. Targeted patient education that is tailored to patient needs, allows for two-way feedback between the patient and doctor and has been shown to improve BP control [5–7]. Whether there are gaps in patient knowledge about BP management that could be addressed via patient education for Australian adults is not known.

We conducted a survey within the Australian general public to: (i) identify content needs to support the control of high BP and; (ii) preference for delivery of information related to managing high BP. Given that general practitioners (GPs) are the medical professionals that predominantly manage high BP in the Australian community, a group of GPs were also surveyed to understand their information preferences to support high BP management.

## Methods

### Study protocol

The community survey included basic demographic questions including age range, sex, postcode, education level and employment status. In addition, the community survey included questions related to high BP control and preferences for accessing information related to high BP. Participants from the Australian general public were also asked if a doctor had previously told them they had high BP, if they were currently taking medication for high BP and how often they take their BP medication.

The GP survey was sent to currently practising GPs and included questions on GP demographics, concerns for BP management, currently used information sources, and preferred formats for education and information dissemination about high BP.

The survey questions were developed by the investigator team and revised based on feedback from colleagues including those with qualitative research experience. In addition, the survey questions were piloted among a general public consumer representative and a GP. The surveys were delivered online and promoted via email and social media to members of the Australian public via the High Blood Pressure Research Council of Australia (HBPRCA), the Australian Cardiovascular Alliance memberships, Kidney Health Australia, Heart Foundation, and Stroke Foundation communication channels between November 2020 and April 2021. Anonymous data were collected using REDCap, a secure online web application. This project was approved by the Alfred Hospital human research ethics committee (HREC number 630/20), and all participants provided informed consent to take part in the study.

### Participant recruitment

The survey was promoted to the general public and general practitioners via social media via generic invitation (see Supplementary material 1) from November 2020 to January 2021. Supporting organisations (Australian Cardiovascular Alliance, Kidney Health Australia, Heart Foundation and Stroke Foundation) supported the promotion of the survey via social media using a snowball method of tagging, liking and sharing posts. Three social media posts were shared for the general public survey and two posts were shared for the GP survey between November 2020 and February 2021. A preliminary analysis of responses was undertaken in December 2020 with a final generic invitation posted to social media in February 2021 (no additional responses were received from the final invitation). In addition, Primary Health Networks were contacted to share the invitation to GPs through their networks. Both surveys were closed by April 2021. Neither general public nor GP participants were offered any incentive to complete the survey.

### Content needs related to BP control

To identify knowledge gaps to inform content needs, participants from the general public were asked to select their concerns related to high BP using a Likert scale ranging from “most concern” through to “least concern”. GPs were asked to select the area of most concern related to BP control using a Likert scale ranging from ‘no concern’, through to “very concerned”. GPs were also asked to select areas that they would like more information related to BP management (e.g., the relationship between BP thresholds and individual risk factors). See Supplementary Table 1 for the questions used in both surveys.

### Preferences for accessing information related to BP management

Participants from the general public were asked to select all sources they currently use to access information related to BP including social media, family or friends, their doctor and other health organisations. These participants were also asked to select all sources that they would prefer to access information related to BP management in the future. GPs were asked to select the information sources that they were most likely to use for BP control according to a Likert scale ranging from ‘most likely’, to ‘least likely’ and to select all formats they would prefer to access information in the future (Supplementary Table 1).

### Statistical analysis

Statistical analyses were performed in Stata version 16.1 (StataCorp, USA). Frequencies were completed to summarise the responses to the survey. In general, data are presented as number and percentage. GraphPad Prism (version 8) was used for creating the figures.

## Results

### Participant characteristics

Of the 465 participants from the general public, 68% were female, 72% were of middle to older age and 48% were highly educated (Table 1). More than half (58%) of the community participants reported they had previously been diagnosed with high BP and half (49%) were taking medications to manage BP. Interestingly, 98% of those taking BP lowering medications reported that they take their medication ‘as prescribed’ by their doctor. Most participants (50%) were from Victoria, Australia.

**Table 1:** Characteristics of participants from the Australian general public that completed the survey about knowledge of high blood pressure (*n* = 465).

Variable	*N* (%)
Age	
30 years or younger	44 (9)
30–40 years	45 (10)
40–49 years	41 (9)
50–59 years	71 (15)
60 years or older	264 (57)
Female 318 (68)	
Education level	
High school (up to year 12)	105 (23)
Bachelor’s degree	101 (22)
Higher education degree	222 (48)
Other	37 (8)
Employment	
Unemployed*	181 (39)
Permanent or fixed-term employment	161 (35)
Training or studying	19 (4)
Casual employment	26 (6)
Self-employed and/or contractor	78 (17)
Blood pressure history	
Previously diagnosed with high blood pressure	249 (58)
Currently taking medications to manage blood pressure	213 (49)
Takes medications as prescribed by doctor	209 (98)
Location	
Victoria	230 (50)
New South Wales	102 (22)
Queensland	64 (14)
Western Australia	29 (6)
South Australia	27 (6)
Tasmania	10 (2)

*This response may include retired individuals as retired was not a response option so it was not possible to delineate the two group (see supplementary material for questions).

### Key concerns for managing high BP

**Fig. 1:**
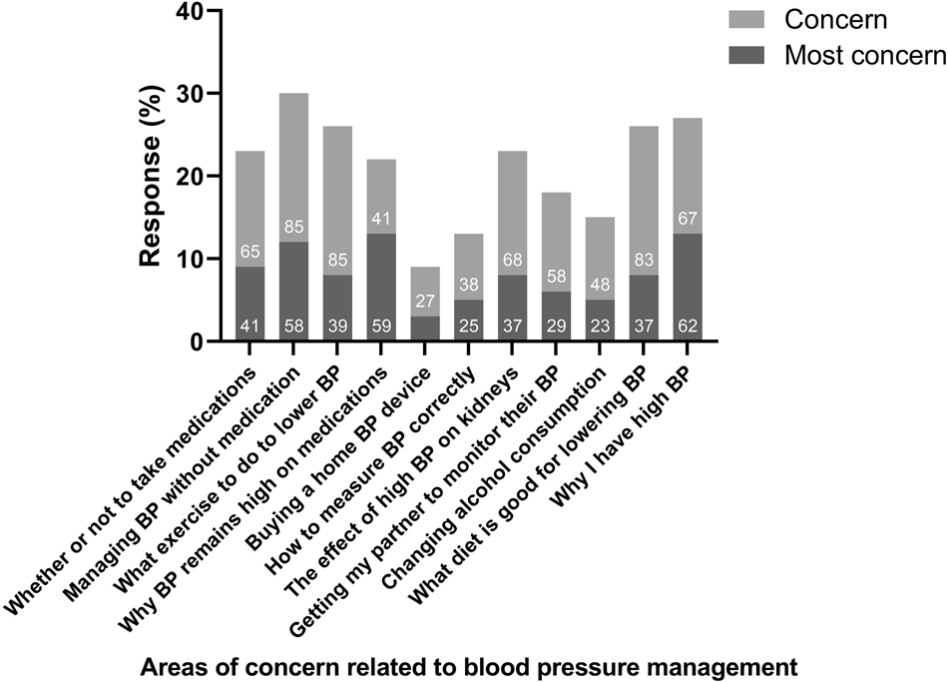
The key concerns relating to blood pressure (BP) management among Australian adults (*n* = 465).

The management of BP without medication was the most common concern raised among the Australian public (Fig. 1). Other areas for concern included ‘why do I have high BP?’, ‘what diet is most beneficial to lower BP?’ and ‘what exercise should I do to manage high BP?’ (27%, 26%, and 26% selected as an area of concern or most concern respectively).

### Preferences for accessing information related to BP

**Fig. 2:**
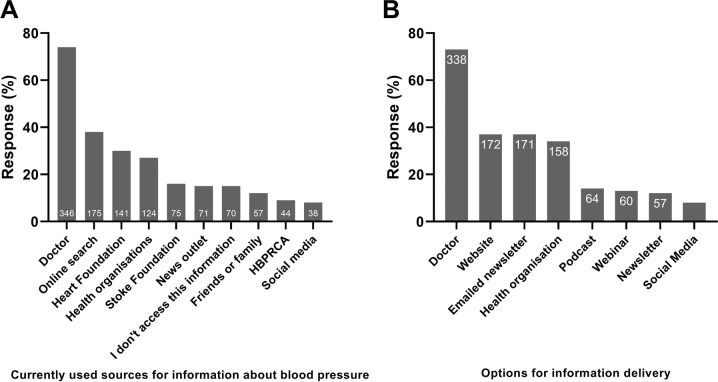
Preferences for accessing information regarding the management of blood pressure among Australian adults (*n* = 465). **A** currently used sources to obtain information about blood pressure and **B** preferences for information delivery. The number of responses were *n* = 39 for social media for the delivery of information regarding the management of blood pressure.

Figure 2 illustrates the currently used and preferred options for information delivery for high BP. Most participants (73%) selected their doctor as the preferred source of information related to BP. A website, an emailed newsletter or other health organisations were the next most popular formats to access information related to high BP (37%, 37% and 34% respectively). Only 8% of participants would consider accessing information regarding BP control via social media.

### GP responses

Demographics of the GP participants (*n* = 23) are reported in Supplementary Table 2. GPs responded that they would like more information related to BP control in elderly patients with dementia (57%, *n* = 13), what to do if either systolic or diastolic BP is high while the other is normal (48%, *n* = 11) and how to manage high BP in physically active/normal weight young people (48%, *n* = 11) (Supplementary Fig. 1). GPs reported they were currently mostly likely to use therapeutic, national, and international guidelines to access information about managing blood pressure (87%, 78% and 78%, respectively). The top preference for information delivery was a one-page summary on BP management for specific groups or topics (57%, *n* = 13, Supplementary Fig. 2)

## Discussion

Our study identified content and delivery preferences for information to optimise BP management among Australian adults, which to our knowledge has not been previously defined. The novel findings included a preference for information centred on lifestyle factors related to BP management and issues around medications. Overwhelmingly, respondents had a preference to access information on BP management from their GPs. This could be achieved by providing GPs with one-page evidence-based summaries of this information that will support patient education to ultimately improve BP control.

The importance of education for patients and health care providers was emphasised by the Lancet Commission on Hypertension to increase the detection of hypertension and effectiveness of BP management [8]. Various forms of hypertension education have been investigated in the past. A multi-faceted education intervention has been shown to reduce all-cause and hypertension-related mortality rate by more than 50% [9]. A broad population-based education strategy achieved a 5–7 mmHg reduction in systolic BP. It is important to note, however, that a recent meta-analysis of education interventions found that different modes of health education delivery had differing efficacy for BP management [10]. While the present study provided novel insight into areas of perceived need and preferences for education delivery by community members and GPs, future work will need to establish the effectiveness of education that is implemented in line with these needs and preferences.

Lifestyle management of cardiovascular risk factors (e.g., via exercise and/or dietary intervention) remains the cornerstone first line therapeutic approach to reduce cardiovascular disease risk related to high BP [11]. Indeed, exercise interventions of various forms have been shown to reduce BP [12, 13], providing equivalent reductions in BP to antihypertensive medication [14], and when combined with pharmacotherapy will have an even greater BP lowering effect [15]. Similarly, there is strong evidence that eating habits that limit the intake of sodium (e.g. Dietary Approaches to Stop Hypertension; the ‘DASH’ diet) will also achieve clinically meaningful BP reductions [16] as well as minimising alcohol intake and maintaining a healthy weight [17, 18]. Despite this evidence, respondents to this survey highlighted a knowledge deficit in lifestyle management including exercise and diet. In addition, participants identified they would like information about how to manage high BP via lifestyle modification, with or without the need for medication. On the other hand, participants identified a preference for education delivery directly from their GP. This highlights a potential gap in patient-GP interactions surrounding lifestyle management of high BP.

An Australian study of GPs identified that although GPs felt qualified to provide patient education on the lifestyle management of high BP, they expressed some negative sentiment towards the effectiveness of such interventions because of a lack of patient enthusiasm, willingness, and commitment to make consolidated behaviour change [19]. Whilst time was reported as a primary barrier to providing effective lifestyle counselling, some GPs surveyed in that study also cited little access to allied health practitioners (namely exercise physiologists and dieticians) who may be able to provide specific and tailored advice. Indeed, referrals to an exercise physiologist and dietician for chronic disease management occur in a minority of GP consultations in Australia (0.1% and 0.4% of GP consultations, respectively) [20, 21].

Given the patient preference for information delivery from GPs, combined with perceived GP barriers to providing this information, alternative methods of education delivery may be required. Although in a small sample, the GP responders to the current survey identified a preference for information delivery via one-page summaries or fact sheets to support BP control. This could be a useful medium to fill the patient need for more information on lifestyle management of high BP in a time efficient manner, whilst also providing direct information on seeking the advice or referral to an allied health practitioner who is an expert in this area. Indeed, as a direct result of this survey, a compendium of one-page infographics has been developed and will be distributed via GP networks and are available on the HBPRCA website https://www.hypertension.org.au/blood-pressure-you/#understanding. It is hoped that this will enhance education and understanding of high BP and its management within the Australian community, and result in meaningful change to the burden of high BP.

## Limitations

In this cross-sectional study of the Australian general public, it was not possible to determine the response rate for the survey due to the recruitment methods used. In addition, the survey was optional and predominantly completed by women and Victorian residents so there may be selection bias in the sample. However, the survey was completed by Australian adults from every state in Australia and predominantly included those aged 40 years or older who are a key demographic for BP management. An additional limitation is the small sample of GPs that were recruited. Due to the recruitment strategies used the sample is not representative of the Australian public or GPs. The nature of snowball sampling is such, that it cannot be considered for a representative sample or in that case for statistical studies. Given that only social media was used to recruit general public participants and that the oldest age range was >60 years, it is not possible to investigate specific preferences among older ages groups. It is possible that older patients taking anti-hypertensive medications and who do not access social media may have been excluded from this survey. A positive of the potential selection bias is that members of the Australian public and GPs with an interest in BP management responded given the high proportion of respondents taking BP medications and targeted recruitment of GPs through the HBPRCA. As such, the findings likely highlight the information content needs and preferences from an engaged audience. Future research is required to determine the utility of the information content and delivery preferences proposed in the study for improving BP control.

## Conclusions

This study identified that Australian adults would prefer more information about the management of BP without medications and via lifestyle education delivered by their GP. In addition, this study identified that GPs would prefer information for optimising BP control to be provided as one-page fact sheets on specific topics. Altogether, supporting GPs with short summaries that address content needs of patients, may assist in improving control of high BP.

## Summary

### What is known about the topic

- Effective patient education achieves more effective blood pressure control when the patient is self-monitoring.
- However, blood pressure control remains poor and the delivery of patient education is varied between providers and settings.
- Management of blood pressure is predominantly undertaken in primary care by general practitioners, so providing resources to general practitioners may support patient education.

### What this study adds

- This study identified that Australia community-dwelling adults want information delivered by their doctor on how to manage blood pressure via lifestyle and without medications.
- General practitioners identified that one-page summaries on specific topics would support patient education for blood pressure management.
- Neither community-dwelling adults nor general practitioners wanted to access information about blood pressure management via social media.

## Supplementary information

Supplementary material

Supplementary Table 1

Supplementary Table 2

Supplementary Figure 1

Supplementary Figure 2

## Data Availability

Data is available from REC on request.
