# Supplementary material for: Content and delivery preferences for information to support the management of high blood pressure
Source: J Hum Hypertens. 2022 Aug 10;38(1):70–4. doi: 10.1038/s41371-022-00723-8 (PMC10803250; doi:10.1038/s41371-022-00723-8)
Supplement: Supplementary file 1 — Supplementary material [file 41371_2022_723_MOESM1_ESM.docx]

**Supplementary material 1. Text used to recruit participants**

The following text was used to promote the survey via social media to the general public:

Post 1:

*Community members are needed to complete a short, anonymous survey to understand knowledge about high blood pressure. The study findings will be used to create educational activities based on areas of concern and preferences of respondents. You can complete the survey* [*here*](https://redcap.baker.edu.au/redcap/surveys/?s=CWWJE4XL8W)*. For more information email: Rachel.Climie@baker.edu.au*

Post 2:

*We want to develop some educational resources related to #highbloodpressure. Please help us by completing this quick survey and circulating widely within your friends and family.*

The below text was used via email to promote the survey to general practitioners that were members via the High Blood Pressure Research Council mailing list:

*Australian general practitioners are needed to complete a short, anonymous survey to understand knowledge about high blood pressure. The study findings will be used to create educational activities based on areas of concern and preferences of respondents. You can complete the survey* [*here*](https://redcap.baker.edu.au/redcap/surveys/?s=CWWJE4XL8W)*. For more information email:* [*Rachel.Climie@baker.edu.au*](mailto:Rachel.Climie@baker.edu.au)
