# Supplementary material for: Content and delivery preferences for information to support the management of high blood pressure
Source: J Hum Hypertens. 2022 Aug 10;38(1):70–4. doi: 10.1038/s41371-022-00723-8 (PMC10803250; doi:10.1038/s41371-022-00723-8)
Supplement: Supplementary file 2 — Supplementary Table 1 [file 41371_2022_723_MOESM2_ESM.docx]

**Supplementary Table 1**. **Questions to determine content and delivery preferences among the Australian public and Australian General Practitioners. Content needs related to BP management among the Australian public were assessed by identifying areas of concern (1A) and among General Practitioners by identifying areas of concern (1B) and topics they were unsure about or would like more information on (1C)**

| **Questions for the Australian Public Survey** | | | | | | | | | | |  |
| --- | --- | --- | --- | --- | --- | --- | --- | --- | --- | --- | --- |
| **Demographic questions** | | | | | | | | | | |  |
| **Age:** <30 years; 30-39 years; 40-49 years; 50-59 years; >60 years. | | | | | | | | | | |  |
| **Sex:** female; male; prefer not to specify. | | | | | | | | | | |  |
| **Education level:** High school (up to year 10); up to year 12; bachelor degree; higher education degree; other. | | | | | | | | | | |  |
| **Employment status:** unemployed; training/studying; casual employment; contractor/sub-contractor; permanent or fixed-term employment; self-employed. | | | | | | | | | | |  |
| **Postcode:** [use to indicate location] | | | | | | | | | | |  |
| **What are your biggest concerns relating to high blood pressure?** | | | | | | | | | | |  |
|  | Most concern | | Concern | | Neutral | | Less Concern | | Least Concern |  |  |
| Whether or not I need to take medication  How to manage blood pressure without having to take medication  How much and what type of exercise I should do  I am taking medication for high blood pressure, but my blood pressure remains high  What type and where to buy a blood pressure device to use at home  How to measure blood pressure at home correctly  Worried about effects on kidneys  Getting my spouse/partner to understand the importance of monitoring their blood pressure  Whether or not I need to change what or how much alcohol I drink  Whether or not my diet is good for my blood pressure  Why I have high blood pressure |  | |  | |  | |  | |  |  |  |
| \| **Where do you currently get information about blood pressure? (select all that apply)** \| \| --- \| \| Facebook or other social media  From my doctor  From my family or friends  Pharmacy or other community health organisations  I search online for information  News such as TV/Radio, The Conversation, newspapers, online news  The Heart Foundation website  The High Blood Pressure Research Council of Australia website  The Stroke Foundation website  I don't search for information related to blood pressure \| | | | | | | | | | | |  |
| **Which of the following sources would you prefer to get information about managing high blood pressure? (select all that apply)** | | | | | | | | | | |  |
| Newsletter  Emailed newsletter  On a website  Webinar  Podcast  On social media such as Facebook or Twitter  My doctor  Pharmacy or other community health group | | | | | | | | | | |  |
| **Question for General Practitioner Survey** | | | | | | | | | | |  |
| **Demographic questions** | | | | | | | | | | |  |
| **Age:** <30 years; 30-39 years; 40-49 years; 50-59 years; >60 years. | | | | | | | | | | |  |
| **Sex:** female; male; prefer not to specify. | | | | | | | | | | |  |
| **Years of practice experience:** <5 years; 5-9 years; 10-19 years; 20-29 years; >30 years | | | | | | | | | | |  |
| **Number of general practitioners and/or practice nurses in practice:** 1-5; 6-10; 11-15 | | | | | | | | | | |  |
| **What are your biggest concerns relating to managing blood pressure in your patients?** | | | | | | | | | | |  |
|  | No concern | Less concern | | Neutral | | Concerned | | Very concerned | |  |  |
| White coat hypertension  Masked hypertension  Having enough blood pressure measurements to decide when to treat hypertension  What the best time of day to take blood pressure is  Prescribing medication  Other factors such as caffeine, smoking or stress increasing blood pressure  Exercise in patients with hypertension/increased cardiovascular risk  Monitoring blood pressure after initiating therapy  Kidney damage or kidney failure  Measuring blood pressure accurately  Managing high blood pressure when the patient has other conditions  Knowing when and how to screen for secondary causes of hypertension |  |  | |  | |  | |  | |  |  |
| **I am unsure and/or would like more information on blood pressure for the following: (select all that apply)** | | | | | | | | | | | |
| Are blood pressure thresholds dependent on individual factors (e.g. age, BMI, sex etc)  What should I do if systolic blood pressure is above high-risk threshold and diastolic is normal or vise versa?  Measurement and management of blood pressure in overweight/obese patients  Managing high blood pressure in physically active / normal weight or young individuals  Managing high blood pressure in patients with diabetes  Managing high blood pressure in the elderly or in those at risk of dementia | | | | | | | | | | | |
| **I would attend or like information about managing high blood pressure in the following format: (select all that apply)** | | | | | | | | | | | |
| Primary care education session  Newsletter  Short videos explaining individual concepts  Emailed newsletter  On a website  Webinar  Podcast  On social media such as Facebook or Twitter  A one-page summary for specific topics (e.g. blood pressure and diabetes) | | | | | | | | | | | |
| \| I would be most likely to access information about managing high blood pressure provided from: \| Most likely \| Likely \| Neutral \| Less likely \| Least likely \| \| --- \| --- \| --- \| --- \| --- \| --- \| \| Australian Medicines Handbook  Colleagues  Facebook or other social media  High Blood Pressure Research Council of Australia  International clinical guidelines [World Health Organisation/International Society of Hypertension]  National Heart Foundation  Other online sources  Primary health network  Therapeutic guidelines  RACGP  Stroke Foundation  Other \|  \|  \|  \|  \|  \| | | | | | | | | | | | |
