# Supplementary material for: Content and delivery preferences for information to support the management of high blood pressure
Source: J Hum Hypertens. 2022 Aug 10;38(1):70–4. doi: 10.1038/s41371-022-00723-8 (PMC10803250; doi:10.1038/s41371-022-00723-8)
Supplement: Supplementary file 3 — Supplementary Table 2 [file 41371_2022_723_MOESM3_ESM.docx]

**Supplementary Table 2. Characteristics of general practitioners that completed the survey about knowledge of high blood pressure in Australia (n=23).**

| **Variable** | **n (%)** |
| --- | --- |
| **Age:** |  |
| 30-39 years | 5 (22) |
| 40-49 years | 6 (26) |
| 50-59 years | 7 (30) |
| More than 60 years | 5 (22) |
| **Sex:** |  |
| Female | 14 (61) |
| Male | 9 (39) |
| **Years of practice experience:** |  |
| Less than 10 years | 6 (26) |
| 10-19 years | 5 (22) |
| 20-29 years | 4 (17) |
| More than 30 years | 8 (35) |
| **Number of practitioners in practice:** |  |
| 1-5 | 5 (22) |
| 6-10 | 11 (48) |
| 11-15 | 7 (30) |
