# Supplementary material for: Content and delivery preferences for information to support the management of high blood pressure
Source: J Hum Hypertens. 2022 Aug 10;38(1):70–4. doi: 10.1038/s41371-022-00723-8 (PMC10803250; doi:10.1038/s41371-022-00723-8)
Supplement: Supplementary file 4 — Supplementary Figure 1 [file 41371_2022_723_MOESM4_ESM.docx]

**
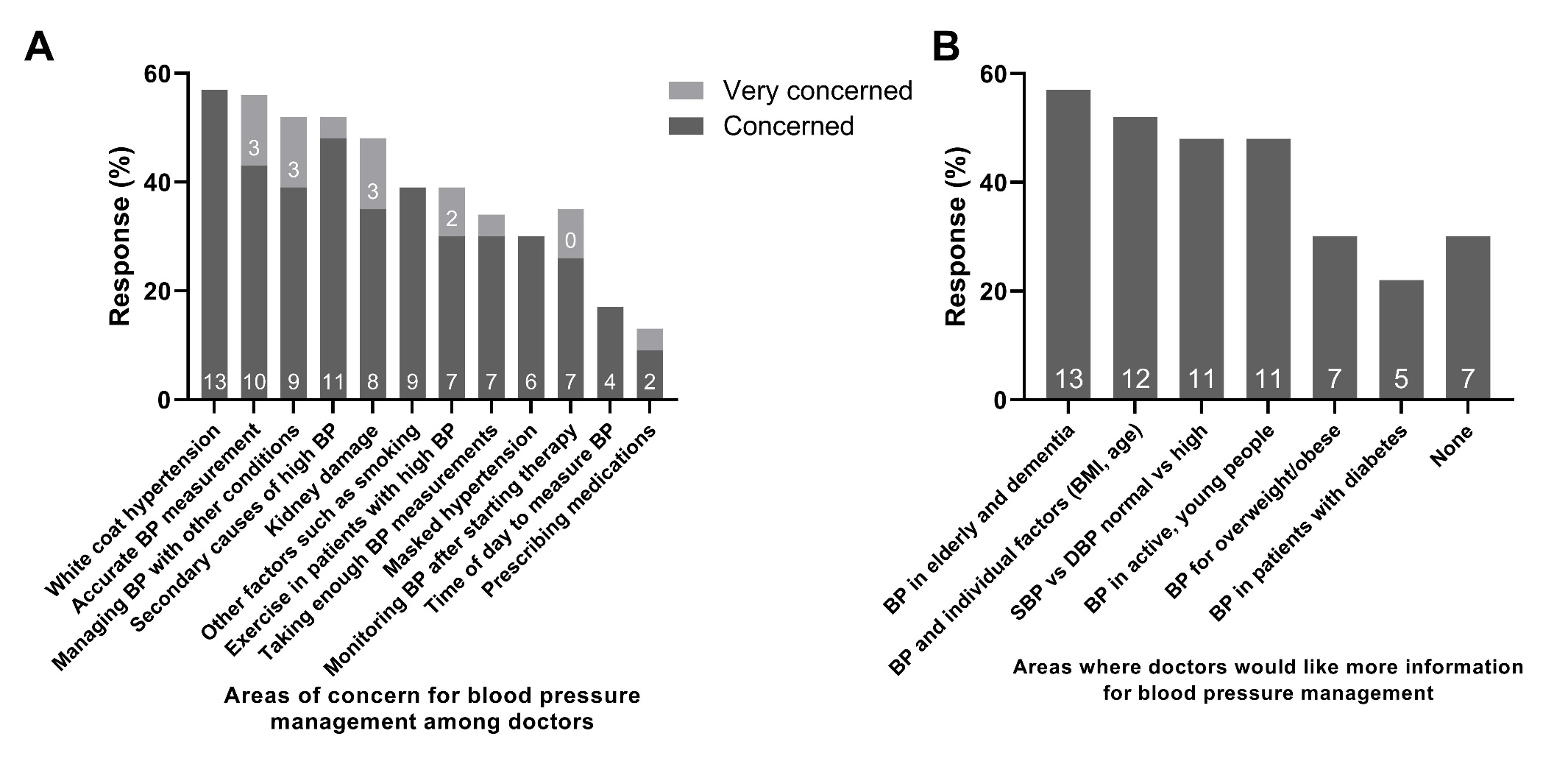
**

**Supplementary Figure 1. The key concerns (1A) and areas where general practitioners would like more information (1B) relating to blood pressure management (n=23) in Australia.**
