# Supplementary material for: Content and delivery preferences for information to support the management of high blood pressure
Source: J Hum Hypertens. 2022 Aug 10;38(1):70–4. doi: 10.1038/s41371-022-00723-8 (PMC10803250; doi:10.1038/s41371-022-00723-8)
Supplement: Supplementary file 5 — Supplementary Figure 2 [file 41371_2022_723_MOESM5_ESM.docx]

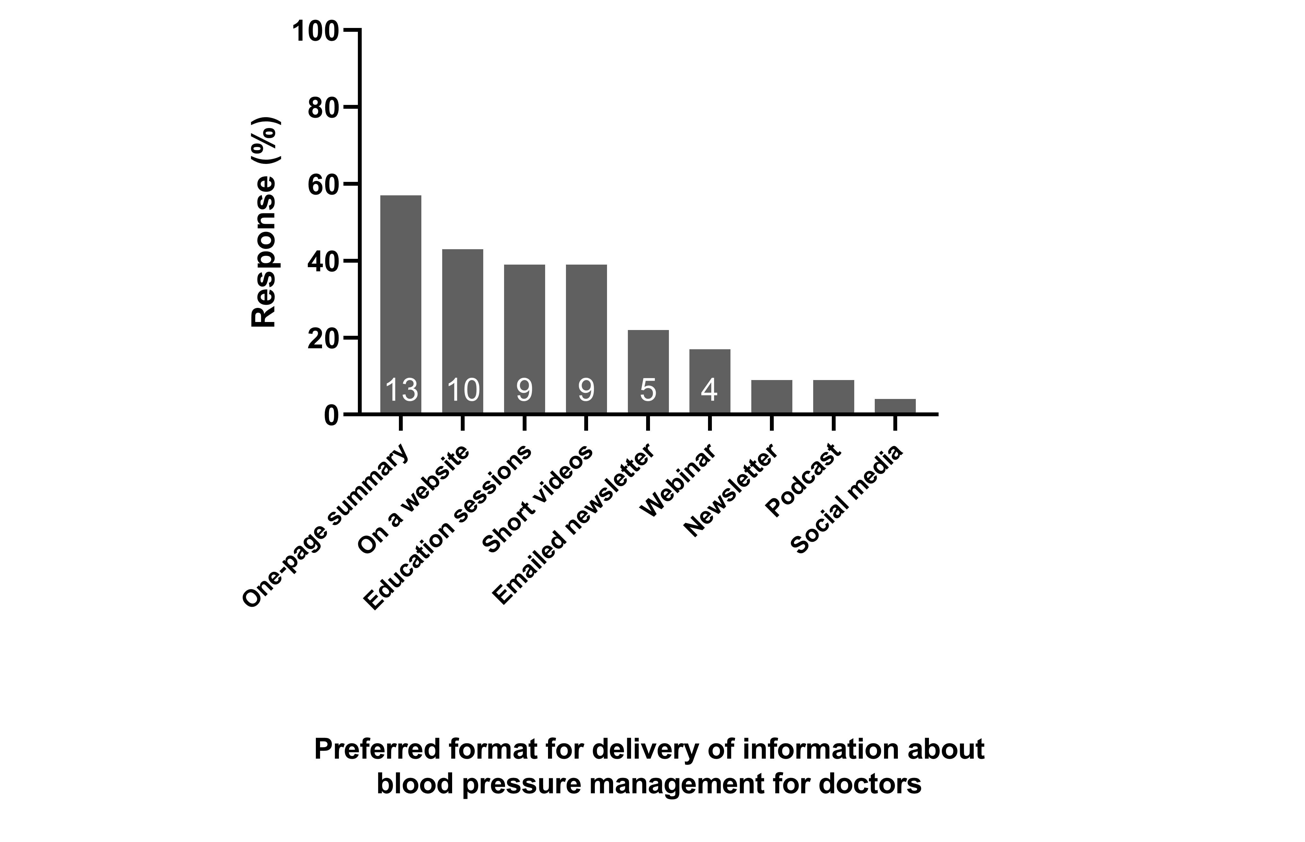


**Supplementary Figure 2. Sources general practitioners (n=23) would prefer to access information for blood pressure management.**
